# More than 10 years after introduction of an acellular pertussis vaccine in infancy: a cross-sectional serosurvey of pertussis in the Netherlands

**DOI:** 10.1016/j.lanepe.2021.100196

**Published:** 2021-09-06

**Authors:** Pauline Versteegen, Guy A.M. Berbers, Gaby Smits, Elisabeth A.M. Sanders, Fiona R.M. van der Klis, Hester E. de Melker, Nicoline A.T. van der Maas

**Affiliations:** a National Institute for Public Health and the Environment, Centre for Infectious Disease Control, Bilthoven, 3720 BA, Netherlands; b Wilhelmina Children's Hospital, Department of Paediatric Immunology and Infectious Diseases, Lundlaan 6, 3584 EA Utrecht, Netherlands

**Keywords:** Bordetella pertussis, cross-sectional, population-based study, seroepidemiological study, pertussis toxin, immunoglobulin G

## Abstract

**Background:**

Pertussis is a respiratory disease and still endemic despite high vaccination coverage. In the Dutch national immunisation programme (NIP) whole cell pertussis (wP) priming vaccines for infants were replaced by acellular pertussis (aP) priming vaccines in 2005. Serosurveillance gives the opportunity to objectively monitor effects of changes in the NIP on infection prevalence and vaccine response in the population over time.

**Methods:**

For this population-based cross-sectional serosurvey a representative sample of Dutch residents (0-89 years) was drawn in 2016/2017. Primary outcome was the percentage of participants with pertussis toxin specific antibody concentrations ≥ 100 IU/ml as an indicator of recent infection, and to identify groups possibly more vulnerable to pertussis infection. Percentages were compared with previous results from 2006/2007.

**Findings:**

In total 7621 persons were included in the analysis. An increase in recent infections from 3•5% to 5•9% was found in the population from 7 years and older (n=6013) in 2016/2017 compared with 2006/2007. Most noteworthy increase was seen in 12-18-year-olds who were wP primed and aP boosted.

**Interpretation:**

Infection prevalence is still increasing in the Netherlands inducing a risk of pertussis disease in vulnerable (age) groups. Delaying the preschool booster might prolong the period of protection during primary school and thereby possibly protect younger siblings. Extra boosters might be considered for risk populations like older adults and people with (pulmonary) co-morbidities, since they have higher chances of complications and hospitalisation.

An unedited Dutch translation of the abstract is available in Supplementary text 1: Nederlandse samenvatting.

**Funding:**

The Dutch Ministry of Health, Welfare, and Sport.

**Research in context**

**Evidence before this study**

Pertussis is a severe respiratory disease. Despite available vaccines and high vaccination coverage, pertussis is still endemic. Two previous serosurveys approximately 10 and 20 years ago described an increase in pertussis infection prevalence from 1% to 3•4% in the Dutch population of 9 years and older. In the meantime, several changes have taken place in the Dutch national immunisation programme, with the most important ones being the switch from whole cell infant priming vaccinations to acellular infant priming vaccinations in 2005 and the addition of an acellular pertussis preschool booster in 2001. We searched PubMed for articles from 2005 up to March 2020 with no language restrictions, using the terms: (sero surveillance OR serosurvey OR seroprevalence OR seroepidemiology) AND pertussis AND population AND cross-sectional. This search revealed several studies from all over the world. Most studies described sero-positivity and some additionally described percentage of recent infection. Peak incidence was mostly in children and adolescents, dependent on age of last vaccination in combination with the epidemic pattern. One study in New South Wales reported results from three subsequent surveys representing different timings of the epidemic cycle: during an epidemic (1997/1998), post-epidemic (2002), and inter-epidemic (2007). Inter-epidemic the proportion of recent infections in the study population was lowest. Material of the study was dependent on residuals of specimens submitted for diagnostic testing. Our study also compares consecutive surveys, all executed in a similar manner with the advantage of a representative sample of the Dutch population and the availability of extensive questionnaire data, e.g. on demographics and vaccination status. The first survey (1995/1996) started before the first outbreak and continued during the first part of the very first outbreak since the introduction of pertussis vaccines in the national immunisation programme. The second survey (2006/2007) started two years after an epidemic and an increase in incidence took place during study inclusion. The current survey (2016-2017) started two years after the last epidemic we have had in the Netherlands.

**Added value of this study**

The here described population-based cross-sectional serosurvey showed an overall increase in pertussis infection prevalence from 3•5% to 5•9% in the Dutch population from 7 years and older during a decade. Whole cell primed adolescents of 12 -18 years not only had the highest percentage of recent infections, but also showed the greatest increase. Acellular primed 7-11-years-olds and whole cell primed 50-64-year-olds showed significant increases as well.

**Implications of all the available evidence**

This study emphasises that priming with either a whole cell or an acellular vaccine is not the only factor involved in the vulnerability for pertussis infection but also in the degree of circulation. Since pertussis infections in vaccinated individuals manifest itself usually mild or subclinical, extra booster vaccinations for school aged children, adolescents, or adults do not seem beneficial to add to the NIP. However, it might be considered to delay the 4 year olds booster with two years to extend the period of vaccine-induced protection and thereby possibly protect younger unprotected siblings. Extra booster doses should be considered only for risk populations like older adults and people with (pulmonary) comorbidities like is common in the Netherlands for the flu vaccine.

Alt-text: Unlabelled box

## 1 Introduction

**Fig. 1:**
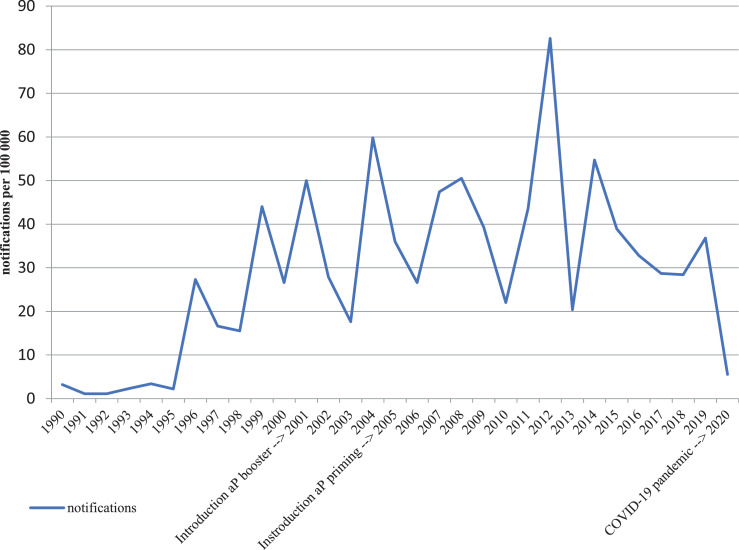
Pertussis notifications

Pertussis is a severe respiratory disease caused by *Bordetella pertussis* and is transmitted between humans by coughing and sneezing [1]. Pertussis presents typically with paroxysmal coughing, inspiratory whooping, and posttussive vomiting and can affect individuals of all ages, although infants are at greatest risk of serious complications [2]. Older adults and people with (pulmonary) comorbidities are also at risk of complications and hospitalisation [3]. Since the start of the national immunisation programme (NIP) in 1957 in the Netherlands with a whole cell pertussis (wP) vaccine, disease incidence and mortality dropped enormously, but from 1996 onwards pertussis epidemics have been observed regularly (Fig. 1). Since then, several changes have been implemented in the NIP.

The most important changes in the NIP with a possible impact on this serosurveillance study were the switch from wP priming in infancy to priming with acellular pertussis (aP) vaccines in 2005 and the addition of an aP booster at 4 years of age in 2001 [4]. The switch from wP to aP priming has been made because of reactogenicity of the Dutch wP vaccine [5]. Moreover, the Dutch wP vaccine had a low estimated vaccine effectiveness since the early 1990s [6]. The aP vaccines in the Dutch NIP contained at least pertussis toxin (Ptx), filamentous haemagglutinin (FHA), and pertactin (Prn) (aP3), and sometimes additionally fimbriae types 2 and 3 (Fim2/3) (aP5). Over the years, different aP3/5 vaccines have been used with various amounts of antigens. Vaccination with aP vaccines provides higher antibody concentrations to the vaccine antigens, but appears to be less protective on the long term [7]. Other adjustments in the NIP were acceleration of the priming schedule from 3, 4, 5 and 11 months of age to 2, 3, 4, and 11 months in 1999 and the implementation of the maternal pertussis vaccination late 2019. An overview of all changes in the Dutch NIP concerning pertussis vaccines is illustrated in Supplementary table 1.

Serosurveillance gives the opportunity to monitor infection prevalence and vaccine response in a population over time, while notification rates are dependent on factors like awareness of disease and the tendency of the public to visit a doctor. In New South Wales three subsequent serosurveys showed a decrease in recent infections over time, representing consecutively a survey during an epidemic, a survey post-epidemic, and a survey inter-epidemic [8]. To monitor the impact of the NIP in the Netherlands at antibody level, several serosurveillance studies were performed over time as well [9,10]. Previously, an increase in infection prevalence -as indicated by a Ptx IgG level ≥ 100 IU/ml- from 1•0% to 3•4% in the population over 9 years of age was observed between 1995-1996 (1996 first epidemic) and 2006-2007 (last epidemic 2004, next epidemic 2008) [11].

In the Netherlands a relatively high vaccination coverage was continually achieved (92-96%) despite the existence of low vaccination coverage (LVC) areas [12]. The country average vaccination coverage for pertussis at one year of age was 95% and 94% during study inclusion in 2016 and 2017, respectively. The LVC areas have a relatively high percentage of vaccination-refusers based on religious grounds [12].

In the current cross-sectional serosurveillance study performed in 2016-2017, we investigated the change in seroepidemiology ten years after the previous serosurvey and whether changes were possibly related to the switch from wP to aP priming in 2005 or to the in 2001 implemented aP booster vaccination at 4 years of age. Other possible influencing factors, like religion, were also analysed.

## 2 Methods

### 2.1 Study design and participants

From February 2016 through October 2017 a national serumbank for cross-sectional population-based serosurveillance studies was established, as previously described [13]. In short, for the NS an age-stratified two-stage cluster sample was drawn from the population register in forty municipalities and an additional sample in nine LVC municipalities, resulting in a NS of 5745 Dutch residents (0-89 years of age) and 1354 persons living in LVC areas. Participants were asked to donate blood, fill in a questionnaire and bring their vaccination certificate. The study was approved by the Medical Ethics Committee Noord-Holland (METC number: M015-022) and designed and conducted in accordance with the guidelines of the Declaration of Helsinki (1996). Written informed consent was obtained from all adult participants and from parents or legal guardians of minors. A summary of the vaccination background of the different age groups can be viewed in Table 1.

**Table 1:** Vaccination background

Age category	Serosurvey 2006/2007	Serosurvey 2016/2017
0 y	aP priming -	aP priming -
1 y	aP priming -	aP priming -
2 y	wP priming -	aP priming -
3 y	wP priming -	aP priming -
4-6 y	wP priming aP booster	aP priming aP booster
7-11 y	wP/aP primed -	aP priming aP booster
12-18 y	wP primed -	wP priming aP booster
19-34 y	wP primed -	wP priming -
35-49 y	wP primed -	wP priming -
50-64 y	wP primed/unvaccinated -	wP priming/unvaccinated -
65-79 y	wP primed/unvaccinated -	wP priming/unvaccinated -
80 + y	N/A	wP priming/unvaccinated -

### 2.2 Serological analysis

Serum IgG concentrations against Ptx (NVI), FHA (Kaketsuken), and Prn [14] were quantified using the fluorescent-bead-based multiplex immunoassay (MIA) as previously described [15,16]. The measurement was performed using a BioPlex 200 combined with BioPlex Manager 6•1 (Bio-Rad Laboratories). To express antibody concentrations in IU/ml, an in-house standard, calibrated on the Pertussis Antiserum (human) 1^st^ WHO International Standard was used. The lower limits of quantification (LLOQs) were 1•0 IU/ml for all antigens. Values below LLOQs were analysed as ½ LLOQ.

### 2.3 Serosurveillance study 2006-2007

Results from the current serosurveillance study were compared with those from the 2006/2007 study, which was conducted in a similar way [10,11]. Briefly, 5,740 participants were included in the NS and 1,518 in the LVC group. IgG-Ptx levels were measured using the same MIA, but were expressed in EU/ml [15]. EU/ml was transformed to IU/ml as earlier described [16]. A summary of the vaccination background of the different age groups can be viewed in Table 1 .

### 2.4 Statistical analysis

Every participant was assigned a sampling weight incorporating the probability of selection and adjustment for age, sex, urbanisation degree, and ethnicity [13]. Participants were divided in age categories, mainly based on differences in vaccination history of the participants of the current study (Table 1). Participants 0-11 years of age received a similar pertussis vaccination schedule but were divided in small age groups to show the presence or absence of vaccination effects. Participants 12-18 years of age received a similar schedule and were therefore taken together. Participants 19 years and older all received the same vaccination schedule and were divided in age groups spanning 15 years.

Primary study outcome is pertussis infection prevalence in the national sample and in different age groups. Participants were divided in three categories based on the level of their Ptx IgG antibody concentration. Serological cut-offs to indicate pertussis infection are not unanimous since there is no known correlate of protection [17]. We used an IgG-Ptx level of 100 IU/ml to be indicative for recent infection in absence of a vaccination in the last few years, which is used as diagnostic cut-off in the Netherlands for pertussis infection using a single serum sample [18]. IgG-Ptx levels 50-100 IU/ml are also shown to enable comparison with studies using a different cut-off [16,19]. Infection prevalence estimates with 95% confidence intervals (95% CI) were calculated in the participant group of 7 years and older based on previously published literature [20,21]. In our study we noticed low proportions of IgG-Ptx ≥ 100 IU/ml in 7 and 8 year olds (Supplementary figure 1) confirming this age limit. Sample size was calculated to estimate infection prevalence with a precision of 2•5% in the NS and 10-15% in age groups spanning at least 5 years [13].

Secondary study outcomes were: 1. A risk analysis for contracting pertussis 2. A comparison between infection prevalence rates and notification rates and 4. A comparison with the sero-survey 10 years agoFor all participants of 7 years and older of the NS and the LVC areas together of the 2016/2017 survey (n = 6013), we determined whether there were risk factors that were independently associated with an increased chance to contract pertussis, i.e. Ptx ≥ 100 IU/ml, using logistic regression analysis. All variables were first tested in a univariate model and variables with a p-value < 0•1 were included in the multivariable model. By stepwise backward selection, variables independently associated with Ptx antibody concentrations ≥100 IU/ml were identified. Participants from LVC areas were divided into two groups based on religion, one group containing the orthodox-reformed individuals (ORI) who (partly) refuse vaccination and the second group containing the non-ORI. Infection prevalence estimates of both groups were compared with the NS. Calculated odds ratios (ORs) were presented with 95% CI. To gain insight in differences between serum infection prevalence and reported disease incidence in the national sample of 7 years and older and per age group in 2016/2017, weighted infection prevalence was compared with the disease incidence rates calculated from mandatory notifications. Comparisons are presented as rate ratios (RR) with 95% CI. GMCs for three pertussis vaccine antigens (Ptx, FHA, and Prn) with 95% CI were calculated to show vaccination effects and to explore trends between the different pertussis vaccine antibody levels within the national sample in 2016/2017. Additionally, a comparison between the 2006/2007 and the 2016/2017 survey was performed using only the results from participants 7-79 years of age, causing slight differences in sampling weight. Comparisons were made between the NS and per age group.

Analyses were performed using Microsoft Excel and SAS version 9•4.

## 3 Role of the funding source

The Dutch government as study sponsor had no role in study design, data collection, data analysis, data interpretation, nor in the writing of the report or in the decision to submit the paper for publication. All authors had full access to all the data in the study and accept responsibility to submit for publication.

## 4 Results

### 4.1 Infection prevalence 2016/2017

Infection prevalence in the national sample ≥ 7 years of age (n=5745) [13] was 5•9% (95% CI 5•3-6•6). Highest proportions were found in the paediatric population divided in 7-11-year-olds (n=414) having an infection prevalence of 8•7% (95% CI 4•5-12•8) and 12-18-years-olds (n=565) 11•5% (95% CI 8•0-15•0). In the adult cohorts infection prevalence was 3•9% (95% CI 2•4-5•4) in 19-34-year-olds (n=1565), 4•7% (95% CI 3•5-5•9) in 35-49-years-olds (n=1252), 5•5% (95% CI 4•0-6•9) in 50-64-year-olds (n=1159), 6•3% (95% CI 4•3-8•4) in 65-79-year-olds (n=931), and 7•1% (95% CI 1•8-12•4) in the 80+ cohort (n=127).

### 4.2 Risk factors for contracting pertussis

In Table 2 all tested risk factors for contracting pertussis, like sex, age, and religion, are listed. Using the multivariable model, it turned out that in 2016/2017 19-49-year-olds had 0•23 times lower odds to contract pertussis compared with 7-11-year-olds, but 12-18-year-olds had 1•55 times higher odds compared with 7-11-year-olds. The model also showed that 7-18-year-olds who have had the 4-year-olds booster, had 0•44 times lower odds to have Ptx antibody concentrations ≥ 100 IU/ml compared with 7-18-year-olds who did not receive the preschool booster. Finally, when a household consisted of more than two members, the likelihood of having Ptx antibody concentrations ≥ 100 IU/ml increased. Although religion was not a significant risk factor, an additional analysis was performed between the NS, ORI, and non-ORI since we have a large cluster of LVC areas, also known as the Bible Belt, covering a substantial part of the Netherlands. This analysis did not reveal any unexpected outcomes (Supplemental figure 2).

**Table 2:** Potential risk factors for pertussis infection prevalence in the population ≥ 7 years of age

	n (%)n = 6013	% Recent pertussis infection (95% CI)	Univariate Crude OR (95% CI)	p-value	Multivariate Adjusted OR (95% CI)	p-value
Sex				**0•04**		
Male	2677 (44•5)	6•7 (5•8-7•6)	Ref.			
Female	3336 (55•5)	5•4 (4•6-6•2)	0•80 (0•64-0•99)			
Age group, years				**0•001**		**0•0004**
7-11	414 (6•9)	8•9 (6•5-12•0)	Ref.		Ref.	
12-18	565 (9•4)	13•1 (10•5-16•1)	1•54 (1•01-2•33)		1•55 (1•02-2•37)	
19-34	1565 (26•0)	4•3 (3•4-5•4)	0•46 (0•30-0•69)		0•23 (0•06-0•85)	
35-49	1252 (20•8)	4•6 (3•5-5•8)	0•49 (0•32-0•75)		0•23 (0•06-0•85)	
50-64	1159 (19•3)	4•8 (3•7-6•2)	0•52 (0•34-0•80)		0•29 (0•08-1•06)	
65-79	931 (15•5)	6•0 (4•6-7•7)	0•65 (0•42-1•01)		0•42 (0•11-1•59)	
80+	127 (2•1)	7•9 (4•1-13•6)	0•87 (0•42-1•81)		0•54 (0•13-2•32)	
Region				0•57		
North	973 (16•2)	6•99 (5•51-8•72)	Ref.			
Midwest	838 (13•9)	5•37 (3•99-7•06)	0•76 (0•51-1•11)			
Mideast	921 (15•3)	5•97 (4•57-7•65)	0•84 (0•59-1•22)			
Southwest	937 (15•6)	5•12 (3•84-6•68)	0•72 (0•49-1•05)			
Southeast	1233 (20•5)	5•76 (4•56-7•17)	0•81 (0•58-1•15)			
LVC	1111 (18•5)	6•30 (4•98-7•85)	0•90 (0•63-1•26)			
aP booster around 4y of age				**<0•0001**		**0•001**
No, but eligible (7-18 year-olds)	157 (2•6)	19•1 (13•5-25•8)	Ref.		Ref.	
Yes, and eligible (7-18 year-olds)	820 (13•6)	9•5 (7•6-11•7)	0•45 (0•28-0•71)		0•44 (0•27-0•70)	
Not eligible (19 years and older)	5013 (83•8)	4•9 (4•4-5•6)	0•22 (0•15-0•33)		1•19 (0•33-4•27)	
Coughing >2w				0•09		
Yes, 0-5m before sampling	1192 (19•8)	7•2 (5•8-8•8)	Ref.			
Yes, 6-11m before sampling	266 (4•4)	7•1 (4•5-10•7)	0•99 (0•59-1•66)			
No	4245 (70•6)	5•4 (4•8-6•2)	0•74 (0•57-0•96)			
Unknown	310 (5•2)	6•8 (4•4-10•0)	0•94 (0•57-1•53)			
Number of contacts				0•8		
≤median number by age group	3113 (51•8)	6•0 (5•2-6•8)	Ref.			
>median number by age group	2900 (48•2)	5•9 (5•1-6•8)	0•97 (0•79-1•21)			
Number of household members				**0•0002**		**0•0099**
1-2	2417 (40•2)	4•4 (3•6-5•3)	Ref.		Ref.	
3-5	2137 (35•5)	6•5 (5•5-7•6)	1•52 (1•17-1•97)		1•59 (1•15-2•20)	
>5	1025 (17•1)	7•6 (6•1-9•4)	1•80 (1•33-2•43)		1•53 (1•04-2•25)	
unknown	434 (7•2)	7•8 (5•6-10•7)	1•85 (1•24-2•77)		1•86 (1•22-2•82)	
Child <4y in the household				0•367		
No	5438 (90•4)	5•8 (5•2-6•5)	Ref.			
Yes	575 (9•6)	6•8 (4•9-9•1)	1•17 (0•83-1•65)			
Underlying disease						
No	1666 (27•7)	5•5 (4•4-6•6)	Ref.			
Yes	4265 (70•9)	6•0 (5•3-6•7)	1•10 (0•86-1•40)			
Unknown	82 (1•4)	14•6 (8•2-23•6)	2•97 (1•55-5•67)			
Priming by vaccination				**0•0049**		
Whole cell vaccination	2071 (34•4)	6•3 (5•3-7•4)	Ref.			
Acellular vaccination	385 (6•4)	9•4 (6•7-12•6)	1•03 (0•75-1•42)			
Unvaccinated or unknown	3557 (59•2)	5•3 (4•6-6•1)	1•06 (0•90-1•24)			
Ethnicity*				0•4749		
1^st^ and 2^nd^ generation Western people, including Dutch origin	5293 (88•0)	5•9 (5•2-6•5)	Ref.			
1^st^ and 2^nd^ generation non Western people	720 (12•0)	6•5 (4•9-8•5)	1•12 (0•82-1•54)			
(Maternal) education level				0•2016		
High	257 (17•2)	3•11 (1•59-6•02)	Ref.			
Middle	355 (23•7)	3•38 (1•94-5•81)	1•24 (0•94-1•63)			
Low	756 (50•6)	3•97 (2•79-5•61)	1•29 (0•98-1•72)			
Unknown	127 (8•5)	3•15 (1•23-7•82)	1•46 (0•93-2•31)			
Religion				0•3702		
Non-orthodox protestant religion or no religion	5719 (95•1)	5•9 (5•2-6•5)	Ref.			
Orthodox protestant religion	294 (4•9)	7•1 (4•6-10•5)	1•23 (0•78-1•95)			

### 4.3 Reported incidence versus infection prevalence

Our study indicates an infection prevalence of 5•9% in the study population from 7 years and older in 2016/2017. Reported incidence percentages of pertussis notifications in the corresponding years showed that 0•029% in the Dutch population from 7 years and older was diagnosed with pertussis in 2016/2017 [4]. Therefore, infection prevalence is approximately factor 200 higher compared with reported incidence (Table 3). Reported incidence and infection prevalence was highest in 7-18-year-olds, discrepancy between infection prevalence and reported incidence was greatest in older adults.

### 4.4 Comparing different pertussis antigens

Reflecting the pertussis immunisation schedule (2, 3, 4, and 11 months, and 4 years of age), geometric mean concentrations (GMCs) of IgG-Ptx in infants showed high levels from 3 months onwards, with a peak at 5 months followed by a steady decrease up to 10 months, and again an increase at 11 months (Table 4). The high IgG-Ptx GMC of the 4-6-year-olds reflect the acellular booster at 4 years of age. The IgG-Ptx GMCs of the 7-11 (GMC 14 IU/ml; 95% CI 12-17 IU/ml) and 12-18-year-olds (GMC 15 IU/ml; 95% CI 12-18 IU/ml) were higher compared with those of the 19-34 (GMC 8 IU/ml; 95% CI 7-9 IU/ml) and 35-49-year-olds (GMC 9 IU/ml; 95% CI 9-10 IU/ml). The pattern observed for Ptx was comparable with the patterns for FHA and Prn with the exception that FHA showed higher GMCs in the population of 50 years and older and Prn was already decreased in the 12-18 year olds.

**Table 3:** Reported incidence of pertussis notifications versus serum infection prevalence

Age category	Incidence of pertussis notifications in the Dutch population 2016/2017 (%)	Infection prevalence in the study population (%)	Rate ratio (95% CI)
**7-11 y**	0•07	8•6	119•9 (82•9-173•5)
**12-18 y**	0•07	11•5	166•9 (128•3-217•2)
**19-34 y**	0•02	3•9	200•7 (148•1-272•0)
**35-49 y**	0•03	4•7	141•6 (107•8-185•9)
**50-64 y**	0•02	5•5	313•8 (244•9-401•9)
**65-79 y**	0•02	6•3	290•8 (220•1-384•2)
**80+**	0•01	7•1	651•8 (377•3-1126)
**Total**	0•03	5•9	200•3 (178•6-224•7)

**Table 4:** Pertussis antigens in the different age groups

Age category	N= 5727 (%)	Ptx GMC (95% CI) in IU/ml	FHA GMC (95% CI) in IU/ml	Prn GMC (95% CI) in IU/ml
0 y	397 (6•9)	37 (30-44)	33 (28-39)	42 (35-53)
1 m	11 (0•2)	2•1 (1•0-4•2)	3•3 (1•2-9•3)	4•1 (2•8-6•0)
2 m	32 (0•6)	5•1 (3•0-8•8)	7•5 (5•0-11)	14 (7•3-25)
3 m	42 (0•7)	60 (36-99)	34 (18-64)	76 (40-145)
4 m	36 (0•6)	58 (40-83)	50 (38-65)	71 (47-107)
5 m	46 (0•8)	88 (67-116)	75 (53-106)	125 (101-154)
6 m	35 (0•6)	56 (44-72)	51 (41-62)	59 (39-91)
7 m	39 (0•7)	45 (32-65)	49 (37-66)	52 (39-68)
8 m	41 (0•7)	34 (23-51)	34 (27-42)	39 (28-54)
9 m	44 (0•8)	39 (27-56)	24 (18-28)	23 (11-50)
10 m	32 (0•6)	26 (18-38)	22 (18-28)	20 (9•0-43)
11 m	39 (0•7)	62 (35-110)	62 (34-111)	72 (33-155)
1 y	105 (1•8)	56 (45-70)	66 (54-82)	91 (67-123)
2 y	69 (1•2)	9•0 (6•5-13)	19 (14-26)	22 (18-30)
3 y	70 (1•2)	9•0 (6•5-13)	25 (16-39)	20 (13-29)
4-6 y	201 (3•5)	28 (24-33)	61 (52-72)	77 (60-100)
7-11 y	324 (5•7)	14 (12-17)	35 (30-41)	29 (25-35)
12-18 y	443 (7•7)	15 (12-18)	33 (29-38)	15 (13-17)
19-34 y	1243 (21•7)	7•9 (7•2-8•6)	20 (19-22)	14 (12-15)
35-49 y	1009 (17•6)	9•4 (8•6-10)	19 (18-21)	10 (8•8-11)
50-64 y	976 (17•0)	12 (11-13)	27 (25-29)	11 (10-13)
65-79 y	784 (13•7)	12 (11-13)	40 (36-44)	10 (9•1-11)
80+ y	106 (1•9)	12 (8•7-16)	30 (24-37)	5•5 (4•1-7•7)

### 4.5 Differences between 2006/2007 and 2016/2017

**Fig. 2:**
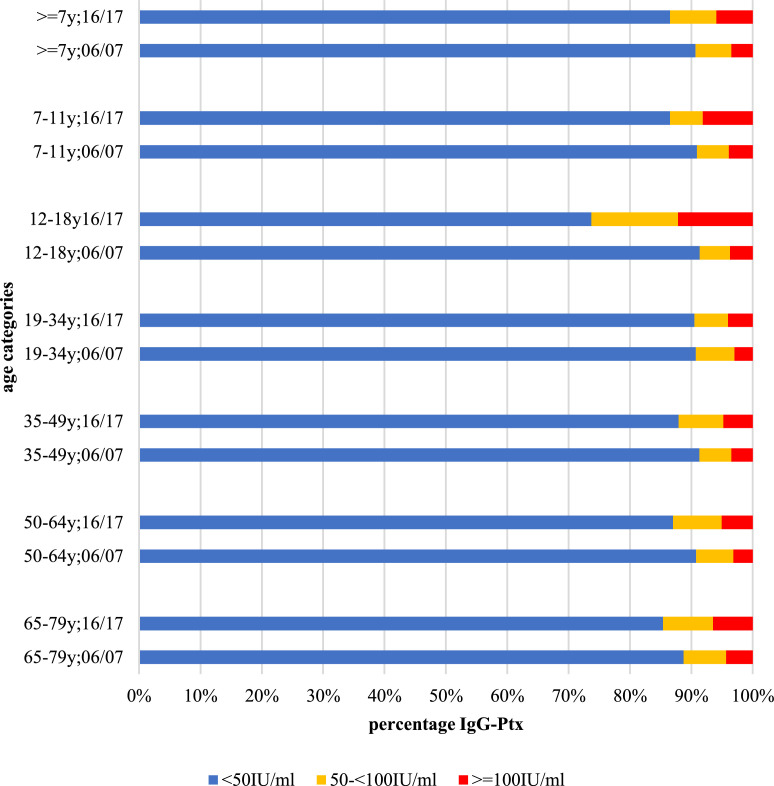
Pertussis infection prevalence in 2016/2017 compared to 2006/2007

A comparison of the proportions of the three IgG-Ptx categories for different age groups between the two studies revealed an overall significant increase of the infection prevalence in the population of 7 years and older (Fig. 2). Previously in 2006/2007 (n=5740) [10] 3•5% showed IgG-Ptx concentrations indicating a recent infection, in the current study of 2016/2017 (n=5745) [13] this percentage increased to 5•9% (p < 0•001). For the different age categories, a significant increase in infection prevalence, varying between 1•6 and 3•3 fold change, was observed in 7-11 years olds (p = 0•012), 12-18 years olds (p < 0•001), and 50-64-year-olds (p = 0•040).

## 5 Discussion

In this 2016/2017 serosurveillance study we observed a still increasing proportion of participants with an IgG-Ptx concentration ≥ 100 IU/ml indicative of recent pertussis infections in the Dutch population of 7 years and older compared with two similar studies one and two decades ago. Most outstanding finding was the increase in IgG-Ptx seroprevalence suggestive for recent infection in the 12-18-year-olds who had received an extra 4-years-olds aP booster compared with their peers in the study ten years earlier.

As previously mentioned, the 12-18-year-olds showed the most striking increase in proportion of recently infected individuals. This seems remarkable since in 2006/2007 this age group was just wP primed and in the current study (2016/2017) they additionally received a preschool aP booster. Some adolescents received one or more aP priming vaccines, but since this was only a small proportion (6•5%) it is not likely to influence the results significantly. Since the introduction of the preschool booster, children become vulnerable for pertussis infection from the age of 7-9 years [20]. The 12-18-year-olds in this 2016-2017 study were 7-14 years of age during the 2012 and/or 2014 epidemics and therefore were vulnerable for pertussis infection at that time. Antibody concentrations induced by infection can reach high levels and are described to decrease approximately with 50% every 7 months, indicating that antibody concentrations can remain high for quite some years [22]. The relative lower GMC for Prn might be explained by the increasing proportion of Prn negative strains in the circulation [23]. At this age, the source of infection is mostly from schoolmates. Considering that all schoolmates are vulnerable around the same age, *B. pertussis* can be easily transmitted between peers during an epidemic.

The increase in the 7-11-year-olds during the decade between the two studies probably reflects a real increase in recently infected individuals [20]. During the 2^nd^ serosurvey, this group was wP primed and a proportion received an aP booster at 4-years of age. In the current study, this group was completely aP primed and aP boosted. Infant priming with an aP vaccine is associated with a higher risk of pertussis later in childhood [7]. As a result, the 7-11 year old children in the current study might be more vulnerable than those from the previous study resulting in a higher infection prevalence. To prolong the duration of vaccine induced protection and thereby possibly also protect unprotected younger siblings in the household, it can be considered to delay the 4-year-olds booster. Serologic and cellular data indicate that the preschool booster can probably be safely delayed with two years [21,24]. In Europe, the timing of the booster varies between 4 and 8 years [25] and infant notification rates do not seem to correspond to the timing of this booster [25,26]. Additionally, aP vaccines seem to protect less against transmission of *B. pertussis* than wP vaccines do [27]. If the increase in infection prevalence is related to the increase in aP primed individuals, we might expect a further increase in the next decade. School aged children tend to have mostly assortative physical contacts of long duration which makes them vulnerable for close-contact infections and therefore they might have a large contribution to *B. pertussis* transmission.

In the adult population, only an increase of recent infections is observed in the 50-64-year-olds. Next to assortative contacts, this age group mixes more with other age groups than younger or older individuals [28]. In this age group, the source of pertussis infection is most often relatives and workplace [29]. The increase might be due to potential contact of middle-aged adults with an increasing proportion of aP primed relatives. From the age of 50 onwards, an increase in FHA GMCs was observed. Since we do not see this increase for other pertussis antigens, this is likely due to infection with other microbes that contain FHA or FHA-like proteins [30].

Next to certain ages and the absence of a preschool aP-booster, a larger household also increased the risk to contract pertussis. Living in a larger household is associated with higher number of contacts and additionally children and adolescents are more likely to live in larger households [28]. Therefore, this risk factor interacts with age and therefore also with vaccination background. LVC areas caused by religious groups that refuse vaccination, did not influence the risk of pertussis as it did for measles and poliomyelitis [31,32]. This might be caused by the effectiveness of the vaccines used. Nor pertussis vaccines nor pertussis infection cause lifelong protection, where measles vaccines tend to protect lifelong. Besides the risk factors discussed in this article, molecular changes on the pathogen level to escape the vaccine are known to have increased the circulation of *B. pertussis* as well [33].

Every surveillance method has its shortcomings, but combining reported incidence with infection prevalence, we can monitor both disease and infection pressure. The latter is important to estimate to risk for vulnerable (age) groups. Discrepancy between reported pertussis incidence in the Dutch population and infection prevalence in this study population was possibly caused by asymptomatic pertussis infection, or atypical presentation of pertussis disease in immunised individuals [34], and by limited awareness of pertussis in the population over 7 years of age. Costs might play a role as well, since in the Netherlands general practitioner visits are covered by insurance, but additional lab diagnostics usually involves costs for the patient. Therefore, general practitioners do not always confirm diagnosis by lab diagnostics and consequently do not report.

Since vaccinated individuals usually only experience mild or no symptoms when they get infected [35], there is only limited benefit of booster vaccinations for the individual. Ptx antibodies obtained by aP vaccination decay in less than 5 years [36]. Vaccinating everyone every 5 years is not cost effective and will probably result in a low vaccination coverage amongst adults, as shown in Austria and France [37]. Therefore, in order to increase herd immunity there is a need for new vaccines which induce long term protection as well as protection against transmission, without increasing reactogenicity. Meanwhile, the focus of the NIP is protecting populations that are at risk of complications due to *B. pertussis* infection, e.g. the very young unvaccinated infants by vaccinating expectant mothers. Maternal vaccination has been implemented in many countries already including the Netherlands late 2019 and has proven to be very effective to protect the very young infants [38]. Next to protecting infants it can be considered to protect other risk groups by offering extra booster vaccines to older adults and people with (pulmonary) co-morbidities like is common in the Netherlands for the flu vaccine.

One of the strengths of this study is the large randomly chosen study population recruited in a relatively small timeframe, which makes it possible to reliably extrapolate data to the general Dutch population. Also, the availability of serum in combination with extensive questionnaires is unique in such a large cohort. A limitation of the study is the cross-sectional design, fluctuations in B. pertussis infection pressure over the years, will cause cohort effects influencing the differences between age groups now and a decade ago. Furthermore, we cannot prove causality between the increased infection related prevalence and the switch from wP to aP vaccination and the introduction of the aP preschool booster, because other factors also changed over time. Other limitations are the stepwise model selection which might give an over-optimistic impression [39], recent infection is arbitrarily defined, and different diagnostic cut-offs are used in different countries. High Ptx antibody concentrations at young age are probably due to vaccination, but infection cannot be ruled out.

In conclusion, despite the switch to aP vaccines and the addition of a preschool aP booster more than 10 years ago, seroprevalence of Ptx antibody levels indicating to recent pertussis infection is still increasing especially in school aged children and adolescents. Since pertussis infection often presents itself mild or even subclinical in vaccinated individuals, extra booster vaccinations for school aged children, adolescents, or adults do not seem beneficial to add to the NIP. However, it might be considered to delay the 4 year olds booster with two years to extend the period of vaccine-induced protection. Extra booster doses might be considered for risk populations like older adults and people with (pulmonary) co-morbidities, since they have higher chance of complications and hospitalisation.

## Contributors

Conceptualisation by PV, NATvdM, GAMB, HEdM, FRMvdK, and EAMS. Luminex data generated by GS. Underlying data verified by GS, PV, GAMB, NATvdM. Data analysis was performed by NATvdM with input from PV, GAMB. PV wrote the first draft of the manuscript and all co-authors contributed to subsequent drafts. All authors read and approved the final manuscript.

## Declaration of interest

None of the authors received payment or service from a third part at any time, nor does anyone have a financial relationship with entities in the bio-medical arena. None of the authors have any patents relevant to the work.

## Appendix Supplementary materials

Image, application 1

Image, application 2

## Footnotes

Supplementary material associated with this article can be found, in the online version, at doi:10.1016/j.lanepe.2021.100196.
