# Supplementary material for: More than 10 years after introduction of an acellular pertussis vaccine in infancy: a cross-sectional serosurvey of pertussis in the Netherlands
Source: Lancet Reg Health Eur. 2021 Sep 6;10:100196. doi: 10.1016/j.lanepe.2021.100196 (PMC8589709; doi:10.1016/j.lanepe.2021.100196)
Supplement: Supplementary file 1 [file mmc1.docx]

# **Caption for supplementary material**

Versteegen et al, TLRHEUROPE 2021

Supplementary text 1: Nederlandse samenvatting

Supplementary figure 1: Proportion of IgG-Ptx in the national sample

Supplementary figure 2: Pertussis infection prevalence in the national sample compared to low vaccination coverage areas

Supplementary table 1: *B. Pertussis* vaccine schedules and vaccines in the Netherlands
