# Supplementary material for: More than 10 years after introduction of an acellular pertussis vaccine in infancy: a cross-sectional serosurvey of pertussis in the Netherlands
Source: Lancet Reg Health Eur. 2021 Sep 6;10:100196. doi: 10.1016/j.lanepe.2021.100196 (PMC8589709; doi:10.1016/j.lanepe.2021.100196)
Supplement: Supplementary file 2 [file mmc2.docx]

**Supplementary text 1: Nederlandse samenvatting**

Achtergrond: Pertussis (kinkhoest) is een luchtweginfectie die ondanks een hoge vaccinatiegraad nog steeds endemisch is. In het Nederlandse rijksvaccinatieprogramma werden in 2005 cellulaire kinkhoest vaccins voor zuigelingen vervangen door acellulaire kinkhoest vaccins. Serosurveillance geeft de mogelijkheid om in de Nederlandse populatie de effecten van veranderingen in het rijksvaccinatieprogramma op de infectieprevalentie en vaccinrespons in de tijd objectief te monitoren.

Methoden: Voor deze cross-sectionele serosurveillance van de Nederlandse populatie is in 2016/2017 een representatieve steekproef van Nederlanders (0-89 jaar) getrokken. De primaire uitkomstmaat was het percentage deelnemers met pertussis toxine-specifieke antilichaamconcentraties ≥ 100 IU/ml, dat geldt als indicator voor een recente infectie en om groepen te identificeren die mogelijk kwetsbaarder zijn voor een pertussisinfectie. Percentages zijn vergeleken met eerdere resultaten uit de serosurveillance van 2006/2007.

Bevindingen: In totaal werden 7621 personen in de analyse betrokken. Bij de bevolking van 7 jaar en ouder (n=6013) werd in 2016/2017 een toename van recente infecties gevonden van 3·5% naar 5·9% ten opzichte van 2006/2007. De meest opmerkelijke toename werd gezien bij 12-18-jarigen die als zuigeling cellulaire kinkhoestvaccins hebben gehad en additioneel een acellulaire voorschoolse booster toegediend hebben gekregen.

Interpretatie: De pertussis infectieprevalentie in Nederland neemt nog steeds toe, wat leidt tot een risico op kinkhoest bij kwetsbare (leeftijds)groepen. Het uitstellen van de voorschoolse booster kan de beschermingsperiode tijdens de basisschool verlengen en daardoor mogelijke jongere broers en zussen beschermen. Extra boosters kunnen overwogen worden voor risicopopulaties zoals ouderen en mensen met (pulmonale) comorbiditeiten, omdat zij een grotere kans hebben op complicaties en ziekenhuisopname.

Financiering: Ministerie van Volksgezondheid, Welzijn en Sport.

**Supplementary figure: 1 Proportion of IgG-Ptx in the national sample**

**Supplementary figure 2: Pertussis infection prevalence in the national sample compared to low vaccination coverage areas**

ORI: orthodox reformed individuals within low vaccination coverage areas; non-ORI: non-orthodox reformed individuals within low vaccination coverage areas; NS: national sample.

**Supplementary table 1: *B. Pertussis* vaccine schedules and vaccines in the Netherlands**

| Year | Scheme | Vaccine composition | Change | pertussis Vaccine name (Company) |
| --- | --- | --- | --- | --- |
| before |  |  |  | No vaccine available |
| 1954 |  | DTwP | Combination vaccine became available | DTwP (RIVM) |
| 1957 | 3,4,5,11 m | DTwP | Start of NIP | DTwP (RIVM) |
| 1962 | 3,4,5,11 m | DTwP-IPV | Addition of IPV | DTwP-IPV (RIVM) |
| 1997 | 3,4,5,11 m | DTwP-IPV + Hib | Addition of Hib tot schedule | DTwP-IPV (RIVM) |
| 1999 | 2,3,4,11 m | DTwP-IPV + Hib | Accelerated vaccination | DTwP-IPV (RIVM) |
| 2001 | 2,3,4,11 m  4y | DTwP-IPV + Hib  DT-IPV + aP3 | Introduction aP booster, 3 Bp components | DTwP-IPV (RIVM)  monovalent aP (GSK) |
| 2003 | 2,3,4,11 m  4y | DTwP-IPV-Hib  DT-IPV + aP3 | Combination vaccine | DTwP-IPV/Hib (NVI)  monovalent aP (GSK) |
| 2005 | 2,3,4,11 m  4y | DTaP3-IPV-Hib  DT-IPV + aP3 | Introduction aP priming, 3 Bp components | Infanrix IPV + Hib (GSK)  monovalent aP (GSK) |
| 2006 (Jan) | 2,3,4,11 m  4y | DTaP5-IPV-Hib DT-IPV + aP3 | 5 Bp components | Pediacel (SP MSD)  monovalent aP (GSK) |
| 2006 (Jun) | 2,3,4,11 m  4y | DTaP5-IPV-Hib + Pneu  DT-IPV + aP3 | Addition of Pneu to schedule; | Pediacel (SP MSD)  monovalent aP (GSK) |
| 2006 (Jul) | 2,3,4,11 m  4y | DTaP5-IPV-Hib + Pneu  DTaP5-IPV | Combination vaccine, 5 Bp components | Pediacel (SP MSD)  Triaxis Polio (SP MSD) |
| 2008 (Feb) | 2,3,4,11 m  4y | DTaP5P-IPV-Hib + Pneu  DTaP3-IPV | 3 Bp components | Pediacel (SP MSD)  Infanrix-IPV (GSK) |
| 2008 (Jul) | 2,3,4,11 m  4y | DTaP3-IPV-Hib + Pneu  DTaP3-IPV | 3 Bp components | Infanrix-IPV + Hib (GSK)  Infanrix-IPV (GSK) |
| 2009 | 2,3,4,11 m  4y | DTaP3/5-IPV-Hib + Pneu  DTaP3-IPV | 3 or 5 Bp components | Pediacel (SP MSD)/Infanrix-IPV + Hib (GSK)  Infanrix-IPV (GSK) |
| 2010 | 2,3,4,11 m  4y | DTaP5-IPV-Hib + Pneu  DTaP3-IPV | 5 Bp components | Pediacel (SP MSD)  Infanrix-IPV (GSK) |
| 2011 (Oct) | 2,3,4,11 m  4y | DT**aP3**-IPV-Hib-HepB + Pneu  DT**aP3**-IPV | Addition of HepB to schedule, 3 Bp components | Infanrix hexa (GSK)  Infanrix-IPV (GSK) |
| 2017 (Jan) | 2,3,4,11 m  4y | DT**aP3**-IPV-Hib-HepB + Pneu  Td**ap3**-IPV | Reduced dose Bp components | Infanrix hexa (GSK)  Boostrix-IPV (GSK) |
| 2018 (Dec) | 2,3,4,11 m  4y | DT**aP5**-IPV-Hib-HepB + Pneu  Td**ap3**-IPV | 5 Bp components | Vaxelis (SP MSD)  Boostrix-IPV (GSK) |
| 2019 (Dec) | Maternal (from 22 weeks gestational age) | Td**ap3** | Introduction ap booster for pregnant women, 3 reduced dose Bp components | Boostrix (GSK) |
| 2020 | 3, 5, 11m  4y | DT**aP3**-IPV-Hib-HepB + Pneu  Td**ap3**-IPV | Revised priming schedule | Vaxelis (SP MSD)  Boostrix-IPV (GSK) |

m=months; y= years; D=diphtheria; T=tetanus; wP: whole cell pertussis; aP: acellular pertussis; IPV: inactivated poliovirus; Pneu: pneumococcal; Hib: Haemophilus influenzae type B; HepB: Hepatitis B; Bp: *Bordetella pertussis*; NVI: Netherlands Vaccine Institute; GSK: GlaxoSmithKline; SP: Sanofi Pasteur; MSD: Merck Sharp & Dohme.
